# Pit Latrines and Their Impacts on Groundwater Quality: A Systematic Review

**DOI:** 10.1289/ehp.1206028

**Published:** 2013-03-22

**Authors:** Jay P. Graham, Matthew L. Polizzotto

**Affiliations:** 1 Department of Environmental and Occupational Health, and; 2 Department of Global Health, George Washington University School of Public Health and Health Services, Washington, DC, USA; 3 Department of Soil Science, North Carolina State University, Raleigh, North Carolina, USA

**Keywords:** groundwater, latrine, privy, sanitation, siting standards, water quality

## Abstract

Background: Pit latrines are one of the most common human excreta disposal systems in low-income countries, and their use is on the rise as countries aim to meet the sanitation-related target of the Millennium Development Goals. There is concern, however, that discharges of chemical and microbial contaminants from pit latrines to groundwater may negatively affect human health.

Objectives: Our goals were to *a*) calculate global pit latrine coverage, *b*) systematically review empirical studies of the impacts of pit latrines on groundwater quality, *c*) evaluate latrine siting standards, and *d*) identify knowledge gaps regarding the potential for and consequences of groundwater contamination by latrines.

Methods: We used existing survey and population data to calculate global pit latrine coverage. We reviewed the scientific literature on the occurrence of contaminants originating from pit latrines and considered the factors affecting transport of these contaminants. Data were extracted from peer-reviewed articles, books, and reports identified using Web of Science^SM^, PubMed, Google, and document reference lists.

Discussion: We estimated that approximately 1.77 billion people use pit latrines as their primary means of sanitation. Studies of pit latrines and groundwater are limited and have generally focused on only a few indicator contaminants. Although groundwater contamination is frequently observed downstream of latrines, contaminant transport distances, recommendations based on empirical studies, and siting guidelines are variable and not well aligned with one another.

Conclusions: In order to improve environmental and human health, future research should examine a larger set of contextual variables, improve measurement approaches, and develop better criteria for siting pit latrines.

An estimated 2.6 billion people lack access to improved sanitation—defined as facilities that hygienically separate human excreta from human contact [World Health Organization (WHO/UNICEF 2010)]. Improved sanitation includes water-based toilets that flush into sewers, septic systems, or pit latrines; simple pit latrines; and ventilated improved pit latrines. There is strong evidence that access to improved sanitation can reduce diarrhea morbidity and mortality as well as soil-transmitted helminths (Albonico et al. 2008; Cairncross et al. 2010b).

The United Nations (UN), through the Millennium Development Goals, has set a target of halving by 2015 the proportion of the population without sustainable access to improved sanitation (WHO/UNICEF 2012c). To achieve this target, approximately 1 billion people in urban areas and 900 million people in rural areas must gain access to improved sanitation by 2015 over the baseline year, 1990 (WHO/UNICEF 2012c). In low-income countries [with a gross national income per capita of ≤ US$1,025 (World Bank 2013)], many households use improved or unimproved pit latrines because of their low cost and availability (Cairncross et al. 2010a; Jain 2011). Improved pit latrines are the most basic and inexpensive form of improved sanitation. They typically consist of a pit—circular, rectangular, or square—dug into the ground and covered with a concrete slab or floor with a hole through which excreta falls. Unimproved pit latrines are those without slabs or platforms.

In concert with sanitation goals, the UN has also set explicit targets to increase the proportion of the global population using an improved drinking-water source (WHO/UNICEF 2012c). In the context of low-income countries, water from improved sources is frequently derived from groundwater via protected springs, protected dug wells, tube wells, and boreholes (UN 2008). Thus, the use of groundwater (which typically receives no subsequent treatment to improve quality) for drinking water supplies is increasing dramatically (Rosa and Clasen 2010).

Because of the increasing uses of both pit latrines and groundwater resources in low-income countries, there is concern that pit latrines may cause human and ecological health impacts associated with microbiological and chemical contamination of groundwater. Pit latrines generally lack a physical barrier, such as concrete, between stored excreta and soil and/or groundwater (van Ryneveld and Fourie 1997). Accordingly, contaminants from pit-latrine excreta may potentially leach into groundwater, thereby threatening human health through well-water contamination. In this study, we assessed the known and measured environmental health impacts associated with groundwater contamination by pit latrines. In particular, we *a*) calculated global pit latrine coverage, *b*) systematically reviewed empirical studies of the impacts of pit latrines on groundwater quality, *c*) evaluated latrine siting standards, and *d*) identified knowledge gaps regarding the potential for and consequences of groundwater contamination by latrines.

## Methods

*Global pit latrine coverage*. We used existing survey data to estimate the percentages of people per country who *a*) use pit latrines for sanitation, *b*) do not have any sanitation facilities, and *c*) use groundwater sources for drinking water [see Supplemental Material, Table S1 (http://dx.doi.org/10.1289/ehp.1206028)]. Data from the most recent reports for each country were obtained from Demographic and Health Surveys (USAID 2012), *Multiple Indicator Cluster Surveys* (UNICEF 2012), and China’s Economic, Population, Nutrition, and Health Survey (WHO/UNICEF 2012a, 2012b). We included improved latrines [flush toilets and toilets that pour/flush to pit latrines (water is poured by hand for flushing), ventilated improved latrines, and pit latrines with slabs] and unimproved latrines (traditional latrines, pit latrines without slabs, and shared latrines) when estimating pit latrine use (see Supplemental Material, p. 2, for definitions of types of sanitation). Composting toilets, considered improved facilities, were not included in our analysis, nor were sanitation facilities for which final disposal of human excreta is unknown (e.g., hanging latrines and bucket latrines). For estimates of the proportions of improved versus unimproved latrines, we assumed that unspecified latrines were split evenly between improved and unimproved. Data for people without a sanitation facility include “no facility” and “open defecation in bush/field.” National survey data do not typically characterize shared facilities because they are considered unimproved sanitation. Therefore, for shared sanitation, we applied the average proportion of facilities that were pit latrines (44%) based on seven national surveys that provided more detailed information (see Supplemental Material, Table S1). Groundwater use comprised both improved and unimproved modes of accessing groundwater, including tube wells and boreholes, protected wells, protected springs, unprotected wells, and unprotected springs, but not centralized water sources that may originate from groundwater.

To calculate the global totals for pit latrine use, we multiplied the country-wide percentages by the UN estimates of 2010 populations (UN 2011) and summed all data presented in Supplemental Material, Table S1 (http://dx.doi.org/10.1289/ehp.1206028). We used our estimate of global latrine use in conjunction with estimated excreta production rates of 1,200 g urine/person/day and 350 g wet feces/person/day for rural developing country settings (Feacham et al. 1983) to estimate daily quantities of urine and feces deposited into latrines.

*Review of studies on groundwater contamination from pit latrines*. To find relevant documents describing groundwater contamination derived from pit latrines, we searched the Web of Science^SM^ (http://webofknowledge.com/), PubMed (http://www.ncbi.nlm.nih.gov/pubmed), and Google (http://www.google.com/) using the following keywords: “pit latrine” AND “groundwater”; “privy” AND “groundwater”; “toilet” AND “groundwater”; “sanitation” AND “groundwater”; “pit latrine” AND “aquifer”; “privy” AND “aquifer”; “toilet” AND “aquifer”; “sanitation” AND “aquifer”; “pit latrine” AND “ground water”; “privy” AND “ground water”; “toilet” AND “ground water”; “sanitation” AND “ground water”; “pit latrine” AND “water quality”; “privy” AND “water quality”; “toilet” AND “water quality”; “pit latrine” AND “well water”; “privy” AND “well water”; and “toilet” AND “well water.” We also searched the resulting reference lists and contacted experts to identify additional articles. To provide a critical review of the literature on the occurrence of microbiological and chemical contaminants originating from pit latrines, we more fully characterized the studies that either directly assessed the fate and transport of contaminants from pit latrines or studies that applied statistical methods to estimate a measure of risk associated with the presence of pit latrines. By synthesizing existing results in terms of siting guidelines for pit latrines and well installation, we identified research gaps that must be addressed in order to make better-informed decisions to protect water quality and safeguard human health.

## Results

**Figure 1:**
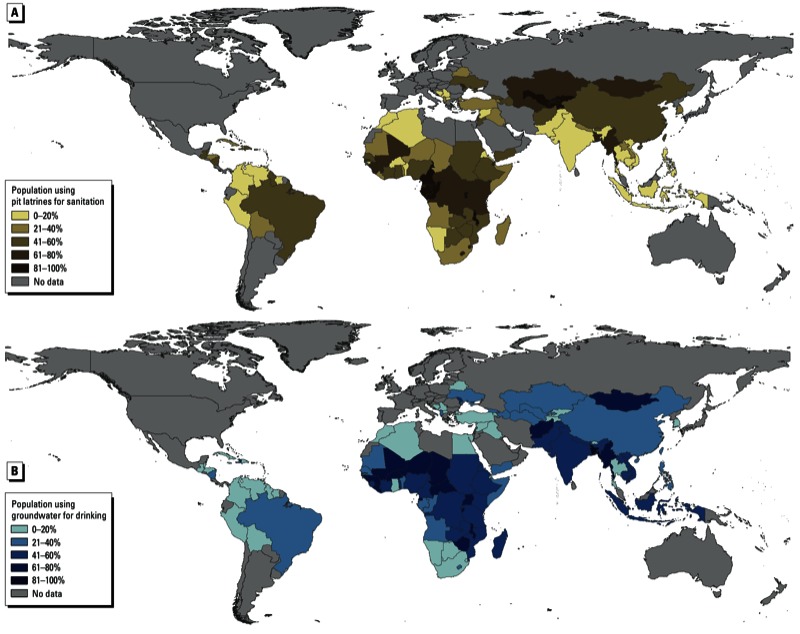
Percentage of low-income country populations using pit latrines as a primary sanitation facility (*A*) and groundwater as a primary drinking water source (*B*). Countries with no data presented were not included in the analysis.

*Global pit latrine coverage*. Globally, there is great variability in latrine coverage. We estimate that approximately 1.77 billion people around the world use some form of pit latrine as their primary means of sanitation [Figure 1; see also Supplemental Material, Table S1 (http://dx.doi.org/10.1289/ehp.1206028)]. In addition, we estimate that 48% of people using pit latrines use facilities characterized as improved, whereas the remainder uses shared or unimproved facilities (e.g., traditional latrines or pit latrines without slabs). The number of users per latrine varies by locale, but based on the excreta production rates of Feacham et al. (1983), globally per day, as much as 2.1 billion kilograms of urine and 0.6 billion kilograms of feces are deposited into latrines. In the countries where pit latrines are prevalent (see Supplemental Material, Table S1), > 2 billion people depend on groundwater for their primary drinking water supply.

These calculations are among the first estimates of the numbers of people using pit latrines and groundwater in low-income countries. Because some national survey data are several years old, estimates have a fair degree of uncertainty at the country level. However, our estimate for the total number of people without any sanitation facility (1.11 billion) is in agreement with the independently calculated Joint Monitoring Program 2010 estimate for open defecation (1.1 billion people) (WHO/UNICEF 2012c), which suggests that our approximations may be more robust at the global level. In addition, our estimate of the total 2010 population for countries included in this analysis (5.22 billion) is consistent with the UN population estimate for “less developed regions” [or “all regions of Africa, Asia (excluding Japan), Latin America and the Caribbean plus Melanesia, Micronesia and Polynesia”] of 5.66 billion (UN 2011).

*Studies on groundwater contamination from pit latrines*. Twenty-four studies directly assessed the transport of contaminants or applied statistical methods to estimate a measure of risk associated with the presence of pit latrines (Table 1); these studies assessed either chemical contaminants (4 studies), microbial contaminants (2 studies), or both (18 studies). Human excreta are the main input to pit latrines, although other inputs may contribute significantly to pit contents depending on local practices [see Supplemental Material, Inputs to Pit Latrines, p. 3, for additional details (http://dx.doi.org/10.1289/ehp.1206028)]. Human feces harbor a large number of microbes, including bacteria, archaea, microbial eukarya, viruses, and potentially protozoa and helminths (see Supplemental Material, Table S2) (Feachem et al. 1983; Ley et al. 2006; Ramakrishna 2007). The largest chemical concerns from excreta disposed in on-site sanitation systems are considered to be nitrate [British Geological Survey (BGS) 2002; Fourie and Vanryneveld 1995; Pedley et al. 2006], phosphate (Fourie and Vanryneveld 1995), and chloride (BGS 2002) (see Supplemental Material, Table S3).

**Table 1:** Summary of selected studies that assessed groundwater or soil contamination associated with pit latrines.^*a*^

Source	Country	No. of latrines in studyb	Experimental design	Subsurface conditions	Sampling time frame	Water quality parametersc	Conclusions
Vinger et al. 2012	South Africa	15	Sampled existing wells	No data	June–July	Ammonia, nitrate, nitrite	Higher levels of contaminants observed at distances < 11 m from pit latrines
Pujari et al. 2012	India	7	Sampled existing wells	Fine loamy silt, sandy loam, intermittent clay	Summer and monsoon seasons	Fecal coliforms, total dissolved solids, nitrate	No to low levels of nitrate and fecal coliforms observed
Banerjee 2011	India	12	Installed test wells	Saturated and unsaturated soils of gravel, sand, silt, clay, and laterite	Premonsoon and monsoon seasons	Total coliforms, fecal coliforms, chloride solution used as tracer	Movement of chloride tracers and coliforms limited to < 10.2 m from pits
Verheyen et al. 2009	Benin	220	Sampled existing wells	No data	Wet and dry seasons, 2003–2007	Adenovirus, rotavirus	Viral contamination of groundwater associated with latrine proximity
Dzwairo et al. 2006	Zimbabwe	3	Installed test wells	Saturated and unsaturated sandy soils	February–May 2005	Ammonia, nitrate, turbidity, pH, conductivity, total coliforms, fecal coliforms	Fecal coliform movement greatly reduced > 5 m from pits; all nitrate levels and 99% of ammonia levels met WHO drinking water standards
Zingoni et al. 2005	Zimbabwe	Not specified	Sampled existing wells and installed test wells	No data	No data	Na, Zn, Cu, Co, Fe, phosphate, nitrate, total coliforms, fecal coliforms	Elevated levels of nitrate and coliform bacteria in most parts of study area
Mafa 2003	Botswana	Not specified	Sampled existing wells	Fractured rock overlain by alluvial sediment, clay, sand, and weathered rock	July and August 2000	Broad set of hydrochemical analyses	Elevated levels of nitrate in several zones where pit latrines were common
Banks et al. 2002	Kosova, Moldova, Siberia	Not specified	Sampled existing wells and springs	No data	1996–2000	Chloride, sulfate, potassium, nitrate	Elevated levels of nitrate likely from latrines
Howard et al. 2003	Uganda	Not specified	Sampled protected springs	Highly variable: clay to sandy soils	Monthly, March 1998 through April 1999	Fecal streptococci, fecal coliforms, nitrate	No significant relationship between microbiological contamination and pit latrine proximity
Still and Nash 2002	South Africa	1	Installed test wells	No data	Bimonthly, 2000–2002	Fecal coliforms, nitrate	Low levels of nitrate (< 10 mg/L) and fecal coliforms (10 cfu/100 mL) found > 1 m of latrine
Ahmed et al. 2002	Bangladesh	Not specified	Sampled existing wells	Two aquifer systems; clay, silt, and fine to coarse sand	2- to 8-week intervals, 1998–1999	Fecal streptococci, fecal coliforms, broad set of hydrochemical analyses	Bacteriological water quality generally good (< 10 fecal coliforms/100 mL); water quality poorly correlated with sanitary surveys
Chidavaenzi et al. 2000	Zimbabwe	2	Installed test wells	Stratified fine-grain sandy soils	Wet and dry seasons	Nitrogen, coliforms	Rapid reductions in coliform, sulfate, and nitrogen levels within 5 m from pits; contamination present up to 20 m
Source	Country	No. of latrines in studyb	Experimental design	Subsurface conditions	Sampling time frame	Water quality parametersc	Conclusions
Jacks et al. 1999	Botswana	4	Sampled existing wells	Well-drained and poorly drained soils	No data	Phosphorous, nitrogen isotopic ratios, chloride	Variable nitrate leaching from pit latrines
Tandia et al. 1999	Senegal	Not specified	Sampled existing wells	Fine to coarse sand	July and November 1989	Broad set of hydrochemical analyses, fecal coliforms	Nitrate contamination in water strongly correlated with latrine proximity
Nichols et al. 1983	USA	8	Installed test wells	3 latrines on clayey soil; 3 on shallow loam; 2 on sand; all soils well-drained	June and August 1975–1979	Nitrate, phosphorus, fecal coliforms	Latrines with peat liners reduced movement of phosphorus and fecal coliforms but not nitrate.
Lewis et al. 1980	Botswana	30 pit latrines in the study area	Sampled existing wells and test wells	Clayey soils and fissured rock	October 1977 through February 1978	Broad set of hydrochemical analyses, E. coli, chloride solution used as tracer	Contamination of wells near latrine with E. coli and nitrate; rapid transport of chloride tracer
Baars 1957	Netherlands	Not specified	Sampled soil and existing wells	Unsaturated sandy soils	September 1951 and January and March 1952	Ammonia, E. coli, nitrate	Contamination in soil samples limited to < 1.5 m from latrines
Dyer 1941	India	1	Installed test wells	Saturated and unsaturated alkaline alluvium soils	December–September	Chloride, nitrate, total coliforms	Movement of total coliforms limited to < 7 m from pit
Caldwell 1938a	USA	3	Installed test wells	Fine gravel to clayey soils	May–November 1933	Bacillus aerogenes, anaerobes, odor, pH, B. coli	B. coli movement limited to 3 m from pits
Caldwell 1938b	USA	1	Installed test wells	Fine gravel to clayey soils	November 1932–November 1933	Nitrate, dissolved oxygen, chloride, nitrite, pH, odor, colon aerogenes group, B. coli, anaerobes	Limited movement of B. coli to 3 m from pit and chemicals to 24 m
Caldwell and Parr 1937	USA	8 bored hole latrines	Installed test wells	Partially saturated fine gravel to clayey soils	May 1932–May 1933	Nitrate, dissolved oxygen, chloride, nitrite, pH, odor, colon aerogenes group, B. coli, anaerobes	Movement of bacteria and chemicals to within 10 m and 26 m of latrine, respectively
Caldwell 1937b	USA	1 envelope pit latrine	Installed test wells	Unsaturated fine gravel to clayey soils	May–November 1933	Colon aerogenes group, pH, odor, B. coli, anaerobes	Bacteria greatly reduced to within 2 m from pit
Caldwell 1937a	USA	1	Installed test wells	Saturated fine gravel to clayey soils	August 1932–November 1933	Colon aerogenes group, pH, odor, B. coli, anaerobes	Movement of bacteria to within 25 m of latrine
Kligler 1921	USA	50	Sampled soil at varying distances	Saturated and unsaturated sand, sandy clay, and clay	Wet and dry seasons, 1918–1919	B. coli, B. aerogenes	Bacterial movement limited to < 5.5 m from pit
Abbreviations: Co, cobalt; Cu, copper; Fe, iron; Na, sodium; Zn, zinc. aOnly studies that either directly assessed the transport of contaminants from pit latrines or studies that applied statistical methods to estimate a measure of risk associated with the presence of pit latrines are included. bNo specific data were provided on the density or number of pit latrines in the study area. cCulture-based assays were used for all microbiological tests, except for Verheyen et al. (2009), who used genotyping methods.							

*Microbiological contaminants associated with pit latrines*. Concentrations of most fecal microorganisms decline after excretion, but these microorganisms may still impair groundwater quality. Several approaches have been used to define the quantities and transport distances of latrine-derived microbial contaminants. The majority of studies that assessed microbiological quality of groundwater in relation to pit latrines applied culture-based assays to measure fecal indicator bacteria (Table 1), including total coliforms, fecal coliforms, and *Escherichia coli* (previously known as *Bacillus coli*), which occur in high concentrations in the feces of healthy adults and have epidemiological evidence to support their use as indicators of water quality (Wade et al. 2003). Caldwell conducted five experimental studies in the 1930s and included the colon aerogenes group and anaerobic bacteria, in addition to *B. coli*, in the analyses (Caldwell 1937a, 1937b, 1938a, 1938b; Caldwell and Parr 1937). Only one study analyzed viruses (adenovirus and rotavirus) to characterize groundwater quality in relation to pit latrines (Verheyen et al. 2009). We found no studies that assessed protozoa or helminths, which typically exhibit little movement in groundwater because of their size (Lewis et al. 1982).

The extent to which microbes from pit latrine wastes may be transported and contaminate groundwater largely depends on the environmental context of the area, particularly hydrological and soil conditions. Nearly half of the studies assessing microbial contaminants used experimental approaches. These studies included either the installation of test wells to measure the quality of water sampled downgradient of pit latrines, the collection of soil samples, or both. Kligler (1921) sampled soil at varying distances from > 50 pit latrines under wet and dry conditions. The maximum distance of bacterial contamination found was 5.5 m from latrines and occurred under wet and sandy soil conditions. Kligler (1921) suggested that a vertical distance of ≥ 3–4.5 m between the bottom of the pit and the water table would maintain safe groundwater quality. In several experimental studies on pit latrines and groundwater, Caldwell (1937a, 1937b, 1938a, 1938b) and Caldwell and Parr (1937) found varying transport distances (ranging from 3 to 25 m) among *B. coli* (i.e., *E. coli*), colon aerogenes (i.e., total coliform bacteria), and anaerobes, depending on the degree of soil saturation and the groundwater flow velocity. In a study of a latrine placed in an alkaline alluvium soil, Dyer (1941) reported that movement of total coliforms was limited to < 7 m from the pit. A relatively short transport distance was also found in South Africa, where high fecal coliform counts [> 10 colony forming units (cfu)/100 mL] were detected only 1 m from a pit latrine (Still and Nash 2002). Dzwairo et al. (2006) found fecal and total coliform contamination greatly reduced > 5 m from pit latrines.

**Figure 2:**
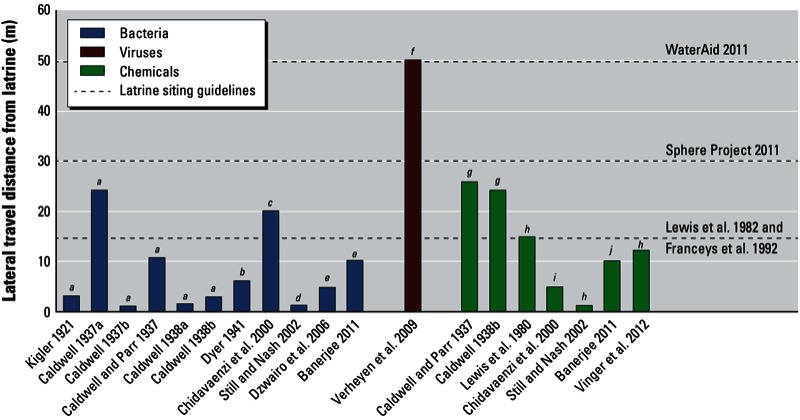
Lateral travel distances of different contaminants emanating from pit latrines in relation to select latrine/water-point siting guidelines. Verheyen et al. (2009) and Vinger et al. (2012) used existing wells to approximate distances, whereas all other studies used test wells to measure distances. *^a^B. coli*; ^*b*^total coliforms; ^*c*^coliforms; ^*d*^fecal coliforms; ^*e*^total and fecal coliforms; ^*f*^adenovirus and rotavirus; ^*g*^chemical stream (nitrate, nitrite, and chloride); ^*h*^nitrate; ^*i*^nitrogen; ^*j*^salt tracer.

In a study of 12 pour/flush latrines, Banerjee (2011) found that transport of total and fecal coliforms increased during the monsoon period and in sandy soils. The author noted that the maximum travel distance of bacteria was 10 m from pits (Figure 2). In contrast, in a study in Zimbabwe, Chidavaenzi et al. (1997) found that groundwater contamination was higher in the dry season than in the wet season, with coliforms detected up to 20 m from a pit.

Nearly one-fourth of the studies analyzed associations between microbiological water quality in preexisting wells and factors such as proximity of pit latrines to assess latrine impacts on groundwater. At a study site in India characterized by a shallow water table and fractured rock aquifer, high concentrations of fecal coliforms were found in domestic wells located near pit latrines and septic tanks (Pujari et al. 2012). At a contrasting site, characterized by alluvial formations, the authors detected no or low levels of fecal contamination (Pujari et al. 2012). In a georeferenced spatial study of viral contamination, Verheyen et al. (2009) sampled 287 drinking-water sources (247 water wells, 25 pumps, and 15 surface water samples) proximate to 220 latrines. Adenoviral DNA was repeatedly detected in 26 water sources, and rotaviral RNA was detected in 1 source. In multiple rounds of sampling, 40 of the 287 drinking-water sources were positive for viral contamination at least once. Verheyen et al. (2009) found a significant positive association between viral contamination of a water source and at least 1 latrine within a radius of 50 m (Figure 2). These authors hypothesized that during the wet season, viruses were transported by groundwater flow in the upper part of the soil, whereas viral transport in the dry season was more likely a result of virus-contaminated surface water.

Associations between groundwater contamination and factors related to sanitation facilities are complicated by the co-occurrence of multiple contaminant sources, particularly when information on groundwater flow patterns is not available. A study of groundwater quality in an informal settlement of Zimbabwe found detectable total and fecal coliforms in more than two-thirds of study boreholes and existing domestic wells (Zingoni et al. 2005). The abundance of pit latrines, used in > 75% of the households, and the presence of informal trading areas within the settlement were likely sources of fecal pollution. The authors suggested that shallow wells and boreholes in the study area, as well as the incomplete lining of most latrines, contributed to high levels of groundwater contamination (Zingoni et al. 2005). In a study conducted in Moldova, Banks et al. (2002) concluded that groundwater pollution within villages was likely caused by latrines, livestock and stored manure, solid-waste landfills, and leakage from wastewater pits.

Even in areas with a high density of pit latrines, microbiological groundwater contamination may not necessarily be detected. Three studies found no strong positive association between poor bacteriological water quality and sanitary surveys or proximity to latrines (Ahmed et al. 2002; Howard et al. 2003; Tandia et al. 1999), although Ahmed et al. (2002) found fecal coliforms and streptococci in sediments 10 m below latrines.

Movement of bacteria from latrines is often limited by formation of a “scum mat,” which develops around the latrine pit and reduces the movement of fecal bacteria (BGS 2002; Caldwell 1937a). This mat (also referred to as a “biologically active layer,” “biolayer,” or “clogged” zone) enhances bacteria removal through filtration and predation by antagonistic organisms, but it may take several months to develop around new latrines (Caldwell and Parr 1937). In addition, clogging may result from blockage of soil pores by solids that have been filtered out, swelling of clay minerals, and precipitation of insoluble salts (Franceys et al. 1992). In a study testing liners as a way to reduce groundwater contamination from pit latrines, Nichols et al. (1983) found fecal coliforms in soil samples taken adjacent to only one of five peat-lined pits, compared with three of three unlined pits. The one peat-lined pit that showed contamination was located in shallow and rocky soil and was under saturated conditions.

*Chemical contaminants associated with pit latrines*. Nitrate. Because of high concentrations of nitrogen in human excreta, its adverse impacts to human health, and its use as an indicator of fecal contamination, nitrate has been the most widely investigated chemical contaminant derived from pit latrines. Consumption of high concentrations of nitrate in drinking water is known to cause methemoglobinemia, and associations with cancer in humans have been observed, although not consistently (Fewtrell 2004; WHO 2011). The WHO-recommended guideline for nitrate in drinking water is 50 mg/L (WHO 2011). Concentrations of nitrate in well water near latrines are highly variable. Although a number of studies that detected total or fecal coliforms did not detect elevated nitrate concentrations in wells (Ahmed et al. 2002; Dzwairo et al. 2006; Howard et al. 2002; Padmasiri et al. 1992; Still and Nash 2002), other studies have reported nitrate concentrations > 100 mg/L (Banks et al. 2002; Girard and Hillaire-Marcel 1997; Lewis et al. 1980; Mafa 2003; Pujari et al. 2012; Tandia et al. 1999). Frequently, groundwater nitrate concentrations near latrines were above local background levels, even if they remained below or near the WHO guideline (Baars 1957; Caldwell and Parr 1937; Chidavaenzi et al. 2000; Jacks et al. 1999; Zingoni et al. 2005).

High nitrate concentrations have been attributed to latrines through association and assumptions based on general proximity, but pinpointing the actual sources of nitrate in groundwater has proved challenging (WHO 2006). Nitrate may be derived from numerous potential sources in urban and rural environments, including latrines, plant debris, animal manure, garbage repositories, livestock pens, soil, and fertilizers (Girard and Hillaire-Marcel, 1997; Howard et al. 2002; Melian et al. 1999; Vinger et al. 2012); and nitrate can be formed and lost through natural soil processes (Jacks et al. 1999). Jacks et al. (1999) used mass-balance calculations to estimate that 1–50% of nitrogen leached to groundwater from latrines in Botswana. Although significant quantities of leached nitrate may have been lost to denitrification in poorly drained soils, the calculations suggested that nitrogen loss from latrines helped describe the high nitrate concentrations of groundwater (50 mg/L) in the area. The authors concluded that moving drinking wells outside of the habituated area would help avoid nitrate contamination of drinking water.

Girard and Hillaire-Marcel (1997) used nitrogen isotopes to determine the source of nitrate pollution in a fractured rock aquifer of Niger. Due to fermentation of feces and ammonia volatilization in latrines, isotopic enrichment of residual matter creates a nitrate source that is isotopically distinguishable from nitrate of other sources. Nitrate concentrations in wells reached 11.6 milliequivalents/L, which may have been a consequence of contamination by latrines and deforestation (Girard and Hillaire-Marcel 1997). The authors cautioned that, given annual population growth rates and increased latrine densities, wells that had safe nitrate concentrations at the time of the study might become polluted in the future.

A more common approach in identifying nitrate sources has been to compare areas with similar environmental characteristics but different population and latrine densities. By analyzing water samples from installed boreholes in an informal settlement in Zimbabwe, Zingoni et al. (2005) demonstrated that the highest nitrate concentrations in groundwater (20–30 mg/L) were associated with the highest population and pit latrine densities of the settlement. In Siberia and Kosova, nitrate concentrations were sometimes > 100 mg/L in groundwater of villages with high latrine densities and minimal septic tanks, but concentrations were below hazardous levels in agricultural and unpopulated settings (Banks et al. 2002). Groundwater nitrate concentrations have also been correlated with proximity to pollution sources, including pit latrines, in Senegal and South Africa (Tandia et al. 1999; Vinger et al. 2012).

Environmental factors also play a role in governing groundwater pollution from latrines. Pujari et al. (2012) compared the impacts of on-site sanitation in two Indian megacities and concluded that hydrogeological conditions were strong predictors of the threat of nitrate contamination of well water; an area with shallow groundwater was more susceptible to pollution from latrines than an area with a deeper water table. In eastern Botswana, buildup of nitrogenous latrine effluent in soils and subsequent downward leaching of nitrate appeared to promote dissolved nitrate concentrations > 500 mg/L in groundwater (Lewis et al. 1980); the authors concluded that the fissured bedrock aquifer allowed for rapid contaminant transport. Whereas soil type immediately below the pit is likely to influence the degree of nitrate transport (Caldwell and Parr 1937), associations with soil type have not always been observed (Nichols et al. 1983). In addition, in an area with high nitrogen loading from latrines but where groundwater was devoid of oxygen, nitrate concentrations were minimal, presumably because of denitrification (Ahmed et al. 2002).

Thus, both environmental conditions and human factors are major drivers of nitrate contamination from latrines, and the highest concentrations in well water are expected to be found downstream of areas with high latrine use (Chidavaenzi et al. 2000; Mafa 2003; Vinger et al. 2012). After nitrate is leached from latrines, a number of factors may control travel distance. Certain chemical contaminants may be transported farther than microbial contaminants because they are not as inhibited by the biolayer that commonly forms around latrines (Caldwell and Parr 1937). Similarly, peat-lined pits were associated with reduced bacterial and phosphate transport from latrines but appeared to be ineffective in limiting nitrate (Nichols et al. 1983). In contrast, Chidavaenzi et al. (2000) estimated that the nitrogen influence from latrines extended only 5 m from the latrine source, whereas microbial contamination extended up to 20 m downstream. In a small study, Padmasiri et al. (1992) observed decreases in soil nitrate concentrations at 1.5 m from the latrine. Overall, although data are sparse, direct measurements and estimates of lateral transport distances for high levels of pit latrine–derived nitrate—where it has been detected—range from approximately 1 to 25 m (Caldwell 1938b; Caldwell and Parr 1937; Chidavaenzi et al. 2000; Lewis et al. 1980; Still and Nash 2002; Vinger et al. 2012) (Figure 2).

Chloride. After nitrate, chloride has been the most commonly investigated chemical indicator of groundwater contamination from latrines because of its high concentrations in excreta and its relative mobility in the subsurface. Although there are no known health risks from chloride in drinking water, concentrations > 250 mg/L may affect the taste and acceptability of water (WHO 2011). In a study from Botswana, Lewis et al. (1980) found the highest chloride concentrations in soils closest to latrines. In Bangladesh, dissolved concentrations reached 400 mg/L at shallow depths, but then decreased with depth and distance from latrines (Ahmed et al. 2002). Chloride is typically transported with minimal retention during groundwater flow, and concentrations frequently track with nitrate levels (Banks et al. 2002; Caldwell 1938b; Caldwell and Parr 1937; Jacks et al. 1999; Lewis et al. 1980; Tandia et al. 1999) unless subsurface conditions promote nitrate reduction (Ahmed et al. 2002). Variable distributions of latrine contaminants resulting from pumping and seasonal fluctuations have been demonstrated by studies using chloride salts as tracers (Banerjee 2011; Lewis et al. 1980).

Ammonia. Ammonia, derived either directly from latrine waste or following denitrification of nitrate released from latrines, has not been reported to accumulate appreciably in groundwater near latrines. In a study of three pit latrines, Dzwairo et al. (2006) observed only one incidence of ammonium (NH_4_^+^) > 1.5 mg/L in well water that was microbiologically contaminated by latrines. In groundwater with latrine-derived nitrate concentrations that exceeded 500 mg/L, Lewis et al. (1980) found NH_4_^+^ at < 0.2 mg/L in all wells but one, which had NH_4_^+^ at 3 mg/L. Similarly, NH_4_^+^ was below the South African National Standard (2 mg/L) in all water samples analyzed by Vinger et al. (2012). Padmasiri et al. (1992) reported that soil concentrations of NH_4_^+^ decreased substantially between 1 and 1.5 m from latrine pits. Ammonia tends to accumulate and persist under anaerobic conditions, and high concentrations are likely when the water table intersects the base of the latrine pit (Ahmed et al. 2002; Baars 1957; Dzwairo et al. 2006).

Other chemicals derived from pit latrines. Nitrite concentrations in well water from near latrines have typically been below drinking water standards (Baars 1957; Vinger et al. 2012), although when present, it has been found in association with nitrate and chloride (Caldwell 1938b; Caldwell and Parr 1937). Phosphate is fairly immobile, and when it was released from latrines, its penetration into soils was minimal (Padmasiri et al. 1992); peat liners further reduced potential transport (Nichols et al. 1983). Accordingly, phosphate concentrations in well water have not been detected at concentrations above water quality standards in association with pit latrines (Zingoni et al. 2005).

Elevated groundwater potassium concentrations may also be derived from latrines, and concentrations have been shown to correlate with those of nitrate and chloride (Banks et al. 2002). The effect of latrines on sulfate concentrations remains unclear, perhaps because of the prevalence of sulfate sources and the number of processes that may remove sulfate from solution in the subsurface. Although Banks et al. (2002) found no evidence that latrines influenced sulfate concentrations in well water, Chidavaenzi et al. (2000) observed increases in sulfate concentrations near latrines during the wet season. Latrines also have been associated with increased well-water turbidity (Dzwairo et al. 2006). Finally, Mafa (2003) measured high concentrations of dissolved organic carbon in wells downgradient of latrines, which might contribute to reducing conditions and elevated dissolved iron concentrations (Zingoni et al. 2005).

## Discussion

*Pit latrine guidelines for mitigating groundwater impacts*. In relation to on-site sanitation, the factors controlling transport of microbial and chemical contaminants in the subsurface have been the subject of several reviews (BGS 2002; Dillon 1997; Gerba et al. 1975; Lewis et al. 1982; WHO 2006), and there is extensive literature that more broadly quantifies contaminant transport processes in groundwater (e.g., Schijven and Hassanizadeh 2000). Soil/rock type, natural and human-altered groundwater flow rates and paths, and the biogeochemical environment of the subsurface all govern contaminant travel distances and rates. Tracking the movement of contaminants is further complicated by microbial die-off and chemical transformations, which may occur heterogeneously over space and time. The potential for widespread groundwater contamination from pit latrines is also affected by social factors, such as latrine use, latrine densities, maintenance, and groundwater pumping. Latrine type, design, materials, and construction quality also influence contaminant containment and leaching from pit latrines. Thus, to effectively evaluate the safety of pit latrine and groundwater source proximity, both environmental and anthropogenic factors must be considered.

Among the studies we reviewed, specific recommendations for minimizing latrine effects on groundwater quality varied. Nichols et al. (1983) suggested that pit liners, such as peat liners, should not be used as a substitute for proper soil conditions, and recommended that latrines not be built in thin, rocky soils. Dzwairo et al. (2006) highlighted the need to *a*) analyze critical parameters such as depth of the infiltration layer and direction of groundwater flow; *b*) develop alternative sanitation options, such as raised or lined pit latrines, to minimize groundwater impacts; and *c*) apply an integrated approach, involving geotechnology and hydrogeology, to solve sanitation problems. Pujari et al. (2012) recommended that latrines be discouraged in rocky areas with shallow water tables. They also suggested that systematic lithological and hydrogeological mapping be conducted and that parameters such as the depth of the water table, soil characteristics, and rock strata be considered prior to installing latrines. Pujari et al. (2012) advised that groundwater sources in areas served by on-site sanitation systems should be monitored by responsible agencies; monitoring should include nitrate, chloride, and fecal coliforms. To minimize the leaching of nitrate, Jacks et al. (1999) suggested *a*) painting latrine ventilation tubes black to increase daytime ventilation rates; *b*) increasing the pH of latrine contents to increase ammonia volatilization; *c*) sealing pits to prevent nitrate leaching and promote denitrification; and *d*) diverting urine for use as a fertilizer for deep-rooted crops. Finally, a number of the studies suggested that pit latrines did not appear to pose a major threat to groundwater quality or public health (Caldwell 1938a, 1938b; Chidavaenzi et al. 2000; Howard et al. 2003; Kligler 1921); this conclusion, which runs counter to general consensus, may have been influenced by the specific latrine siting, environmental conditions, and experimental designs of the studies.

Given the varying transport distances observed for microbiological and chemical contaminants originating from pit latrines (Figure 2), researchers have identified a range of latrine siting guidelines. In their comprehensive review about the risks for groundwater contamination by on-site sanitation sources, Lewis et al. (1982) noted the “traditional” guideline of 15 m as a safe distance between wells and sanitation units. On the basis of statistical associations between latrines and nitrate concentrations in water sources, Tandia et al. (1999) recommended distances of 20 m, 36 m, and 48 m for pits that are in use for < 1 decade, 1–2 decades, and > 2 decades, respectively. Banks et al. (2002) suggested that pit latrines should be located no less than 15–30 m from groundwater abstraction points and should terminate no less than 1.5–2.0 m above the water table. Banerjee (2011) concluded that, with the exception of fissured rock, the safe distance between a pit latrine and water source is 10 m. Vinger et al. (2012) suggested that wells are likely to be contaminated if pit latrines are < 12 m away.

Countries and development agencies often have siting standards for latrine construction. In Haiti, for example, latrines must be sited at least 30 m from any surface water source or drinking water source, and the bottom of the pit must be at least 1.5 m above the maximum height of the water table (Reed 2010). South Africa’s groundwater guidelines recommend that pit latrines are located at least 75 m from water sources (Still and Nash 2002). The WHO suggests minimal risk of groundwater pollution where > 2 m of relatively fine soil exists between a pit and the groundwater table, assuming fill rates are < 50 L/m^2^/day (Franceys et al. 1992). Furthermore, 15 m is suggested as the safe lateral separation between pit latrines and the groundwater supply; this distance can be reduced if the well is not directly downgradient of the pit (Franceys et al. 1992). However, in a more recent and conservative recommendation that seeks to account for a wide variety of contexts, WaterAid (2011) suggests that latrines and water sources should be at least 50 m apart (WaterAid 2011). For disaster response situations, the Sphere Project (2011) has recommended 30 m as a minimum standard for the lateral distance between on-site sanitation systems and water sources, although this value could be adjusted based on the nature of subsurface features.

Overall, threats to groundwater quality from on-site sanitation can be mitigated through technology design, risk assessment, development of protection zones, and monitoring (Lawrence et al. 2001; Lewis et al. 1982; Robins et al. 2007). For septic systems and more complex on-site sanitation technologies, manuals and siting guidelines are widely accessible (e.g., U.S. Environmental Protection Agency 2002), and technology choices generally depend on the available land area for drain fields and vertical separation to the water table. Step-by-step strategies for site-specific analyses of safe sanitation options appropriate for low-income countries have been outlined by the BGS (Lawrence et al. 2001). The BGS guidelines provide a set of rules for determining the optimum horizontal separation between sanitation facilities and drinking-water sources for a variety of hydrogeological environments. These guidelines have been tested in Bangladesh (Ahmed et al. 2002), Uganda (Howard et al. 2003), and Argentina (Blarasin et al. 2002) and have been advocated as sensible practice for aquifers for which data are limited and therefore do not otherwise lend themselves to conventional vulnerability assessment (Ahmed et al. 2002; Blarasin et al. 2002; Howard et al. 2003).

*Moving forward*. Pit latrine and groundwater usage are prevalent in a rapidly growing segment of the world population. Given that approximately 1.11 billion people currently have no sanitation facility [see Supplemental Material (http://dx.doi.org/10.1289/ehp.1206028)], pit latrine coverage is expected to increase as people attempt to move up the sanitation ladder from open defecation to basic sanitation (WHO/UNICEF 2012c). Our analysis of existing literature reveals five key knowledge gaps that could be addressed to improve our understanding and management of groundwater contamination from pit latrines.

Siting latrines in relation to wells. Groundwater flow paths are among the most important factors controlling contaminant transport from latrines to water points. In many areas, the subsurface flow pattern is unknown. Groundwater flow models are needed to better define the limits of chemical transport and pathogen dispersion (Pedley et al. 2006), particularly for complex groundwater systems such as fractured rock aquifers. It is often difficult to determine whether a contamination source is a pit latrine or animal waste and agricultural sources; better assessment of groundwater flow conditions will enable identification of dominant contaminant sources. In locations where horizontal separation of latrines and water points is not possible (e.g., routinely flooded regions), vertical separation has been promoted (Lawrence et al. 2001), but such siting guidelines are not well defined. An improved understanding of contaminants leaching from pit latrines and the transport pathways involved is needed particularly for managing sanitation in densely populated areas, such as refugee camps and informal settlements, as well as areas with rapidly growing populations. Siting guidelines need to consider population pressures and the potential for increased groundwater abstraction, which will alter transport distances and rates.

Understudied and emerging contaminants. To date there has been a focus on a limited number of contaminants that may be found in human excreta. Microbiological monitoring has primarily relied on fecal indicator bacteria, whereas nitrate has been the focus of most chemical studies. In a recent study of groundwater in rural Bangladesh, Ferguson et al. (2012) noted that culture-dependent fecal indicators were not always able to predict total bacterial pathogens. Pit latrine additives are used to reduce pit contents, odor, and insect problems, but little research exists on their makeup or the prevalence of their use (Buckley et al. 2008). Organic chemical contaminants, including endocrine disruptors and pharmaceuticals, that may be excreted in urine and feces and may persist in the environment have not been investigated proximate to pit latrines, but they should be quantified and their potential for transport needs to be assessed. There has also been little research on disposal of other chemicals, such as lime, pesticides, and cleaning agents, into latrines. Finally, it remains unclear whether effects of latrine wastes on the geochemical environment of groundwater may increase downstream contamination. For instance, excreta contains high quantities of organic carbon (Feacham et al. 1983), and plumes of carbon from latrines may promote reducing conditions within groundwater (Mafa 2003), leading to reductive release of trace elements associated with native aquifer materials (Harvey et al. 2002).

Global climate change. Global climate change is widely recognized as a threat to the safety and reliability of drinking water and sanitation supplies, particularly in low-income countries (WHO 2009; World Bank 2012). To date, no studies have specifically addressed these threats in relation to pit latrines and groundwater quality. Many sprawling urban slums, as well as poor rural communities, are currently situated in coastal zones that are flood prone or have high groundwater tables, especially in East Asia (Djonoputro et al. 2010). Rising sea levels will increase the prevalence of flooding and slowly raise groundwater levels, limiting the ability for safe vertical separation between latrine pits and the saturated zone. Over shorter time periods, escalation of storm intensities will increase the probability that groundwater tables will rise above the bottoms of pits at some point during the year; thus, it is likely that contaminant transport from pit latrines to groundwater will increase. Flooding will also likely undermine efforts to increase access to basic sanitation. Urban planning and housing development programs will need better estimates of the potential effects of climate change on on-site sanitation, as well as additional information to determine appropriate sanitation facility designs for different target populations.

Improved sanitation technologies. Technological upgrades to pit latrines may substantially reduce microbiological and chemical threats to groundwater quality. Latrine liners can minimize seepage of pit contents to groundwater, and raised latrines may help minimize groundwater contamination by increasing vertical separation and promoting aerobic digestion of waste (Dillon 1997; Dzwairo et al. 2006; Nichols et al. 1983). Urine-diverting toilets, painted ventilation tubes, and chemical amendments to latrines can minimize nitrate formation and release to groundwater (Jacks et al. 1999). Composting toilets and ecological sanitation technologies may reduce microbial risks and minimize chemical leaching from pit latrines (Dillon 1997; Endale et al. 2012). However, it remains unclear whether these alternative systems are affordable and culturally acceptable to poor populations in low-income countries (Mariwah and Drangert 2011).

Balancing risks. Despite the potential for groundwater contamination, pit latrines remain an important strategy for improving human excreta disposal. These systems are the most basic option for low-income countries to decrease rates of open defecation and increase access to improved sanitation. An intensive effort is needed to develop more robust—yet viable—approaches to siting pit latrines and water sources. Proposed guidelines should be tested empirically to ensure protection of groundwater quality after implementation under local conditions.

## Conclusions

We estimate that approximately 1.77 billion people around the world use pit latrines. This number is expected to increase as populations grow and countries strive to meet the Millennium Development Goals. The use of groundwater as a primary drinking-water source is also increasing. Accordingly, there is a growing need to understand how pit latrines may adversely impact groundwater quality and human health.

Despite the widespread global reliance on both pit latrines and groundwater, we found a limited number of studies that have explicitly examined links between groundwater pollution and contamination from pit latrines. Within these studies, the quality of experimental techniques and chosen indicator contaminants varied greatly. In multiple studies conducted near the same location, there were substantial differences in transport distances of microbiological and chemical contaminants (Caldwell 1937a, 1937b, 1938a, 1938b; Caldwell and Parr 1937). Nevertheless, based on available reports, researchers who looked for groundwater contamination from pit latrines frequently detected it, and studies observed travel distances of up to 25 m, 50 m, and 26 m for unsafe concentrations of bacteria, viruses, and chemicals, respectively (Caldwell 1937b; Caldwell and Parr 1937; Verheyen et al. 2009). Although these contaminant transport distances could potentially be exceeded under certain conditions (e.g., in fractured rock aquifers), most studies of pit latrine–derived contaminants actually showed transport distances that were less than half of the maximum values. Areas with shallow groundwater and areas prone to flooding present the greatest risks, because vertical separation is required between the base of latrine pits and the saturated zone.

The ability to make informed decisions about water and sanitation options is largely inhibited by a scarcity of data, especially regarding the influence of environmental conditions on potential contamination. Guidelines are available for site-specific assessment, and general procedures for siting latrines with respect to water points are common (Lawrence et al. 2001). However, recommendations for mitigating groundwater impacts can be both qualitatively and quantitatively variable, and it remains unclear whether these guidelines can be implemented under local conditions. Many countries already face serious developmental challenges and may not have the resources—human and economic—to implement guidelines (Robins et al. 2007).

In general, siting guidelines vary greatly, and it is often unknown which (if any) empirical studies were used to derive the guidelines. Thus, there is a need to empirically test the effectiveness of specific guidelines under a variety of conditions in order to better merge pit latrine siting guidelines with realistic groundwater threats. Given the scale of pit latrine use, future studies must examine additional contaminants beyond standard indicators, monitor temporal changes in water quality parameters, and evaluate alternative technologies. In addition, efforts are needed to better understand the effects of population pressures and climate change in order to make more informed decisions that optimize latrine and groundwater use and improve environmental and human health.

## Supplemental Material

(188 KB) PDF
