# Supplementary material for: Pit Latrines and Their Impacts on Groundwater Quality: A Systematic Review
Source: Environ Health Perspect. 2013 Mar 22;121(5):521–30. doi: 10.1289/ehp.1206028 (PMC3673197; doi:10.1289/ehp.1206028)
Supplement: (188 KB) PDF [file ehp.1206028.s001.pdf]

Environmental Health Perspectives doi:10.1289/ehp.1206028

Supplemental Material

Pit Latrines and Their Impacts on Groundwater Quality: A Systematic Review

Jay P. Graham and Matthew L. Polizzotto

Table of Contents

Global Pit Latrine Use Calculations and Data……………………………………………2

Glossary defining different types of sanitation..…….……………………………………2

Inputs to Pit Latrines…………………………………………………………..............3

Supplemental Table S1. Summary data for pit latrine use, no sanitation facility, and groundwater use by country………………………………………………………………5

Supplemental Table S2. Summary of selected microorganisms found in human feces of healthy or infected individuals……………………………………………………........8

Supplemental Table S3. Major chemical contents of human excreta…………………......9

Supplemental Material References…………………………………………………...10

Global Pit Latrine Use Calculations and Data for Figure 1.

We used existing survey data to estimate the percentages of people per country who 1) use pit latrines for sanitation, 2) do not have any sanitation facilities, and 3) use groundwater sources for drinking water (Table S1). Total country-wide data were used, and when there was an option, we used percentages of people rather than households. Data were obtained from *Demographic and Health Surveys* (USAID 2012), *Multiple Indicator Cluster Surveys* (UNICEF 2012), and China's *Economic, Population, Nutrition, and Health Survey* (WHO/UNICEF 2012a).

Data and methods of analysis are summarized in the main text. In addition:

- For calculations of percentages of improved vs. unimproved latrines, traditional/rudimentary latrines were qualified as unimproved, shared latrines were qualified as unimproved, and unspecified latrines were split evenly between improved and unimproved.
- Data for people without a sanitation facility include "no facility" and "open defecation in bush/field".
- We used the most recent reports for each country from the consulted sources. The majority of data are from 2005 and later. There is general agreement in data from different sources for a given country, provided report dates are within ~5 years.

Glossary defining different types of sanitation (WHO/UNICEF JMP)

Improved Sanitation

- *Flush toilet* – excreta is flushed into a pit, septic tank, or sewer.
- *Piped sewer system*
- *Septic tank*
- *Flush/pour flush to pit latrine* – toilet that uses water poured by hand for flushing.
- *Ventilated improved pit latrine (VIP)* – waterless pit latrine ventilated by a pipe that extends out of the pit to above the latrine roof. The system aims to reduce odor and flies.
- *Pit latrine with slab* - waterless pit latrine covered by a slab or platform fitted with a hole or seat. The slab must be a cleanable surface and adequately cover the contents of the pit.
- *Composting toilet* – a waterless toilet into which carbonaceous materials are added to the excreta and special conditions maintained to treat human biosolids.

Unimproved Sanitation

- *Flush/pour flush to elsewhere* – excreta may be flushed to street, yard/plot, open sewer, ditch, drainage way or other location
- *Pit latrine without slab* – lacks slab, platform or seat covering hole; often an open pit
- *Bucket latrine* – bucket or other container used for the collection of excreta; disposal location unclear
- *Hanging latrine* – toilet built over body of water; excreta goes drops directly into water
- *No facilities, bush or field* – includes excreta deposited on the ground and covered; excreta wrapped and thrown into solid waste; and defecation directly into surface water.

Inputs to Pit Latrines

Human excreta is the main input to pit latrines, although other inputs are common, such as anal cleansing material, menstrual blood and sanitary napkins, and solid refuse, which may contribute significantly to pit contents depending on local practices (Bhagwan et al. 2008; Buckley et al. 2008; Still 2002). Pit latrine additives used to reduce pit contents, odor or insect problems have been noted in research, but little research exists on the make-up of the additives or the prevalence of their use (Buckley et al. 2008). The volume of water added to the pit can be large if water is used for anal cleansing or flushing in the case of pour flush latrines, or if there is a habit of bathing in the same place as the pit latrine (Chaggu 2003). Depending on the construction of the latrine, water may also enter the pit after a rain event. Most assessments of inputs into pit latrines, however, have focused almost solely on the addition of human excreta. The volume and content of excreta produced by humans varies, and is affected by age, diet, nutrient uptake, climate and the occurrence of diseases associated with infection by pathogenic bacteria, viruses, protozoa and helminths. A review of human excreta production estimated that in developing countries urban adults on average produce 250 grams of feces (80% wet weight), while rural adults produce 350 grams of feces (85% wet weight) (Feachem et al. 1983). The review estimated that 1.2 liters per person per day, was the average amount of urine produced for both rural and urban individuals in developing countries (Feachem et al. 1983). An analysis of pit latrine contents found the solids content range to be 2.0 – 4.2 percent solids (Pescod 1971).

Microbiological Contents of Human Excreta

Human feces harbor a large number of microbes, including bacteria, archaea, microbial eukarya, viruses, and potentially protozoa and helminths (Feachem et al. 1983; Ley et al. 2006; Ramakrishna 2007) (Table S2). It is estimated that the human feces contains 10^{14} microorganisms per gram of dry weight fecal matter, most of which are non-pathogenic (Zhang et al. 2006). Urine has typically been thought to be sterile until it comes into contact with the urethra during urination, but there is evidence that both viruses and viable but non-culturable bacteria may be present in urine in the urinary tract (Anderson et al. 2004; Rodrigues et al. 2007).

Bacteria. In healthy adults, bacteria make up approximately 55% of fecal solids, and 400 to 500 different bacterial species are typically represented in the feces (Ramakrishna 2007). Bacterial flora vary individually and diversity has been found to increase with age (Blaut 2002; Moore and Moore 1995; Ramakrishna 2007). The majority of the bacterial flora belong to three groups: *Clostridium coccoides-Eubacterium rectale* group, *Clostridium leptum* group and *Bacteroides-Prevotella* group (Ramakrishna 2007). Depending on disease patterns among households and communities, pathogenic bacteria may or may not be present in the feces entering pits.

Viruses. Human feces contains a large number of viruses, although relatively few studies have characterized viral diversity in human feces and most viruses are still unknown (Kim et al. 2011; Mansour et al. 2003; Wandell and Wade 2003; Zhang et al. 2006). In a recent study of healthy adults, Kim et al. (2011) found that the concentration of viruses in fecal samples ranged from 1.1×10^8 to 1.5×10^9 viruses per gram of feces (wet weight), whereas bacteria ranged from 3.9×10^9 to 7.6×10^9 bacteria per gram of feces (wet weight) (Kim et al. 2011). Viral pathogens isolated

from the feces of patients with gastrointestinal infections are mostly RNA viruses including rotavirus, enteroviruses, astrovirus, calicivirus, hepatatis E virus, coronavirus and torovirus, and certain serotypes of the enteric adenovirus (Zhang et al. 2006). Table S2 provides a list of the microbiota commonly found in human excreta, as well as some pathogenic microorganisms, and their density.

Menstrual blood may also be disposed of into pit latrines. Thus, there is the potential that blood-borne pathogens, which may include hepatitis B (HBV), hepatitis C (HCV) and human immunodeficiency virus (HIV), may enter the pit. Although blood-borne viruses have been found to persist for more than one week on inanimate surfaces, no data exists on their survival in pit latrines (Casson et al. 1997; Johnson et al. 1994; Kramer et al. 2006).

Chemical Contents of Human Excreta

The chemical composition of urine and feces is highly variable due to factors including diet, drinking water composition, climate, occupation, age, and state of health After water, organic matter makes up the largest component of both feces and urine (Table S3), though this does not immediately cause a chemical risk to groundwater. The largest chemical concerns from excreta disposed in on-site sanitation systems are considered to be nitrogen (BGS 2002; Fourie and Vanryneveld 1995; Pedley et al. 2006), phosphorus (Fourie and Vanryneveld 1995), and chloride (BGS 2002). Most nitrogen is excreted as urea, which readily degrades to ammonium; under aerobic conditions, ammonium will subsequently be microbially oxidized to nitrite and nitrate (Pedley et al. 2006), mobile species in groundwater which can cause methemoglobinemia when consumed in high quantities (WHO 2011). The majority of nitrogen in excreta is found in urine (Table S3), and although large quantities of nitrogen may be deposited to latrines each year, threats to groundwater may be substantially minimized by urine diversion (Drangert 1998; Jacks et al. 1999). Chloride and phosphorus are also predominantly excreted through urine (Table S3) (BGS 2002; Schouw et al. 2002). Chloride is fairly mobile in groundwater and can impact the acceptability of drinking water. Phosphorus, as phosphate, is not a direct health threat from drinking water and is relatively immobile, but high concentrations may promote algal blooms and it is therefore a concern as a contaminant of surface water.

In addition to major chemical components of excreta, there are a number of potential organic and inorganic contaminants found in highly variable concentrations within excreta (Fourie and Vanryneveld 1995). There is a growing concern of pharmaceuticals, household cleaners and personal care products in water supplies, though these are typically of more concern in wet sanitation and centralized systems that have a more complex mixture of chemicals (WHO 2006). Caffeine is also increasingly prevalent in excreta, but it is not frequently detected in groundwater (WHO 2006). Metals, such as lead and cadmium, are predominantly excreted in feces (Schouw et al. 2002) and may provide a residual source of contaminants in latrine sludge.

Supplemental Material, Table S1. Summary data for pit latrine use, no sanitation facility, and groundwater use by country.

Country	Data Source[a]	Report Year	Pit Latrine Use for Sanitation (%)	No Sanitation Facility (%)	Groundwater Use for Drinking (%)	2010 Population[b]
Afghanistan	MICS	2010-2011	42.5	17.7	62.9	31411743
Albania	DHS	2008-2009	24.0	0.0	20.8	3204284
Algeria	MICS	2006	2.2	4.9	13.4	35468208
Angola	MICS	2001	31.0	38.7	37.5	19081912
Armenia[c]	DHS	2010	30.4	0.0	2.5	3092072
Azerbaijan	DHS	2006	58.8	0.3	25.7	9187783
Bangladesh	DHS	2007	60.1	7.5	90.8	148692131
Belarus	MICS	2005	27.5	0.0	12.5	9595421
Belize	MICS	2006	40.7	2.1	7.5	311627
Benin	DHS	2006	11.7	69.5	38.6	8849892
Bhutan	MICS	2010	52.7	3.4	1.5	725940
Bolivia	DHS	2008	25.7	28.4	7.9	9929849
Bosnia and Herzegovina	MICS	2006	2.5	0.1	15.7	3760149
Botswana	MICS	2000	57.2	16.2	2.9	2006945
Brazil	DHS	1996	42.2		21.3	194946470
Burkina Faso	MICS	2006	1.0	62.6	68.9	16468714
Burundi	MICS	2005	92.7	3.0	69.0	8382849
Cambodia	DHS	2010	5.7	55.3	50.0	14138255
Cameroon	MICS	2006	82.6	7.5	49.9	19598889
Central African Republic	MICS	2006	75.4	22.3	69.1	4401051
Chad	DHS	2004	24.3	74.1	77.2	11227208
China	CHS04	2004	49.9	2.3	20.1	1341335152
Colombia	DHS	2010	0.7	4.8	3.2	46294841
Comoros	MICS	2000	95.0	0.7	7.9	734750
Congo	DHS	2005	84.4	10.2	30.6	4042899
Côte d'Ivoire	MICS	2006	42.7	34.0	51.8	19737800
Cuba	MICS	2010-2011	25.7	1.0	18.2	11257979
Democratic People's Republic of Korea	MICS	2009	37.4	0.0	10.8	24346229
Democratic Republic of the Congo	MICS	2010	80.0	14.5	59.1	65965795
Djibouti	MICS	2006	73.4	3.9	2.7	888716
Dominican Republic	DHS	2007	47.6	36.0	9.7	9927320
Egypt	DHS	2008		0.4	4.0	81121077
Eritrea	DHS	2002	9.0	74.3	45.2	5253676
Ethiopia[c]	DHS	2011	56.0	38.2	50.6	82949541
Gabon	DHS	2000	92.8	3.0	40.0	1505463
Gambia	MICS	2005-2006	80.6	4.4	41.0	1728394
Georgia	MICS	2005	57.8	0.0	25.7	4352244

Supplemental Material, Table S1 (cont.)

Ghana	MICS	2010-2011	56.6	0.5	0.1	24391823
Guatemala	DHS	1998-1999	40.9	13.2	14.9	14388929
Guinea	DHS	2005	67.2	30.3	60.7	9981590
Guinea-Bissau	MICS	2006	4.0	31.1	79.1	1515224
Guyana	DHS	2009	43.3	1.0	3.5	754493
Haiti	DHS	2005-2006	32.0	0.0	45.0	9993247
Honduras	DHS	2005-2006	39.8	16.7	15.8	7600524
India	DHS	2005-2006	12.9	56.2	58.6	1224614327
Indonesia	DHS	2007	3.8	8.1	52.3	239870937
Iraq	MICS	2006	28.9	2.5	3.4	31671591
Jamaica	MICS	2005	77.2	0.6	3.3	2741052
Jordan	DHS	2009	43.5	0.0	0.0	6187227
Kazakhstan	MICS	2006	62.3	0.0	22.5	16026367
Kenya	DHS	2008-2009	67.3	14.5	41.4	40512682
Kyrgyzstan	MICS	2005-2006	82.0	0.1	10.1	5334223
Lao People's Democratic Republic	MICS	2006	31.7	50.1	48.7	6200894
Lesotho	DHS	2009	66.8	35.6	36.2	2171318
Liberia	DHS	2007	20.1	54.7	76.2	3994122
Madagascar	DHS	2008-2009	35.1	43.7	53.3	20713819
Malawi	DHS	2010	84.9	9.9	75.3	14900841
Maldives	DHS	2009	27.8	1.0	1.3	315885
Mali	DHS	2006	60.2	19.6	69.9	15369809
Mauritania[c]	MICS	2007	35.1	45.5	37.7	3459773
Mongolia	MICS	2005	67.1	13.4	60.7	2756001
Montenegro	MICS	2005-2006	7.7	0.3	9.2	631490
Morocco	DHS	2003-2004	1.7	15.9	16.3	31951412
Mozambique	MICS	2008	52.7	41.8	55.9	23390765
Myanmar	MICS	2009-2010	74.9	7.0	73.6	47963012
Namibia	DHS	2006-2007	11.7	53.4	16.6	2283289
Nepal[c]	DHS	2011	21.0	38.4	46.9	29959364
Nicaragua	DHS	2001	59.1	13.9	25.3	5788163
Niger	DHS	2006	21.5	78.0	74.3	15511953
Nigeria	MICS	2007	58.9	27.7	47.6	158423182
Pakistan	DHS	2006-2007	13.7	28.4	55.6	173593383
Peru	DHS	2011	1.9	12.0	6.6	29076512
Philippines	DHS	2008	11.7	9.6	39.0	93260798
Republic of Moldova	DHS	2005	62.5	0.0	56.0	3572885
Rwanda[c]	DHS	2010	96.6	1.1	59.0	10624005
Samoa[c]	DHS	2009	10.0	0.1	3.8	183081

Supplemental Material, Table S1 (cont.)						
Sao Tome and Principe	DHS	2008-2009	23.1	57.7	5.9	165397
Senegal	DHS	2010-2011	57.1	16.5	27.6	12433728
Serbia	MICS	2010	4.5	0.0	8.5	9856222
Sierra Leone	MICS	2010	63.4	28.9	54.1	5867536
Somalia	MICS	2006	37.2	53.8	25.6	9330872
South Africa	DHS	2003	36.7	8.1	3.8	50132817
Sudan	MICS	2000	55.2	32.4	40.7	43551941
Suriname	MICS	2006	19.6	6.3	3.1	524636
Swaziland	MICS	2010	69.7	15.4	19.3	1186056
Syrian Arab Republic	MICS	2006	18.2	1.0	7.7	20410606
Tajikistan	MICS	2005	85.5	0.4	14.2	6878637
TFYR Macedonia	MICS	2005	6.9	3.1	7.0	2060563
Thailand	MICS	2005-2006	1.4	0.8	12.3	69122234
Timor-Leste	DHS	2009-2010	28.0	35.8	48.7	1124355
Togo	MICS	2010	31.5	55.7	55.2	6027798
Trinidad and Tobago	MICS	2006	15.0	0.1	1.2	1341465
Turkey	DHS	2003	22.8	0.5	7.3	72752325
Turkmenistan	DHS	2000	71.3	0.6	22.9	5041995
Uganda	DHS	2006	66.4	11.8	73.5	33424683
Ukraine	DHS	2007	47.2	0.0	28.0	45448329
United Republic of Tanzania	DHS	2010	78.8	15.9	48.3	44841226
Uzbekistan	MICS	2006	87.4	0.0	20.1	27444702
Vanuatu	MICS	2007	77.3	3.2	22.7	239651
Venezuela	MICS	2000	6.7	4.4	2.1	28979857
Viet Nam	MICS	2010-2011	18.2	6.4	43.7	87848445
Yemen	MICS	2006	42.1	21.4	35.9	24052514
Zambia	DHS	2007	57.1	23.5	47.1	13088570
Zimbabwe[c]	DHS	2010-2011	42.6	28.3	64.0	12571454

[a]**Data Sources.** MICS: Multiple Indicator Cluster Surveys, UNICEF (http://www.childinfo.org/mics.html); DHS: Demographic and Health Surveys, USAID (http://www.measuredhs.com); CHS04: Economic, Population, Nutrition, and Health Survey, data accessed from WHO/UNICEF Joint Monitoring Program reports (http://wssinfo.org).
[b]**2010 Population.** United Nations, Department of Economic and Social Affairs, Population Division (2011). World Population Prospects: The 2010 Revision, CD-ROM Edition.
[c]Country for which recent DHS data were used to estimate a latrine component of shared facilities (see main text).

Supplemental Material, Table S2. Summary of selected microorganisms found in human feces of healthy or infected individuals[a].

Microorganism	Genera, Family and/or species (Average number of excreted organisms per gram of feces wet weight)
**Bacteria[b,c]**	*Bacteroides* (10^{7-11}), *Fusobacterium* (10^{9}), *Eubacterium* ($10^{8.5-10}$), *Bifidobacterium* (10^{7-11}), *Lactobacillus* (10^{4-9}), *Clostridium* (10^{3-10}), *Clostridium perfringens* (10^{3-10})[a], *Ruminococcus* (10^{10}), *Peptostreptococcus* (10^{10}), *Enterococcus* ($10^{5.3-10}$), *Escherichia* (10^{8}), pathogenic *Escherichia coli* (10^{8})[a], *Citrobacter* (10^{8}), *Enterobacter* (10^{8}), *Proteus* (10^{8}), Klebsiella (10^{8}), *Campylobacter jejuni* (10^{8})[a], *Shigella* (10^{6-7})[a], *Vibrio cholerae* (10^{6-7})[a], *Salmonella typhi* (10^{8})[a], Other Salmonellae (10^{8})[a], *Yersinia enterocolitica* (10^{5})[a]
**Virus[b,d,e,f]**	Podophages (10^{7-9}), Siphophages (10^{7-8}), Microphages (10^{6-8}), Myophages (10^{6-7}), Pepper Mild Mottle virus (10^{9})[g], Enteroviruses (10^{6-7})[a], Hepatitis A virus (10^{6})[a], Rotavirus (10^{6}), Norovirus (10^{7-9})[a]
**Protozoa[a]**	*Entamoeba histolytica* (10^{4}), *Cryptosporidium parvum* (10^{7}), *Giardia intestinalis* (10^{6}), *Giardia lamblia* (10^{5})
**Helminths[a]**	*Ascaris lumbricoides (*10^{4}), Hookworms (10^{2}), *Taenia saginata* (10^{4}), *Trichuris trichiura* (10^{3}), *Clonorchis sinensis* (10^{2}), *Diphyllobothrium latum* (10^{4}), *Fasciolopsis buski* (10^{3}), *Schistosoma* (4-40 per mL of urine)

[a]The presence of these pathogenic microorganisms depends on the prevalence of infection in the community
[b]Feachem 1983
[c]Ramakrishna 2007
[d]Kim et al. 2011
[e]Zhang et al. 2006
[f]Ozawa et al. 2007
[g]per gram of feces dry weight

Supplemental Material, Table S3. Major chemical contents of human excreta.

Chemical Contents	Urine Generation (g/person/d)[a]	Feces Generation (g/person/d)[b]	Yearly Loading to Latrine (kg)[c]
N[d]	7.2 – 16.0	2.6 – 7.4	14.3 – 28.7
P[d]	1.2 – 4.2	1.6 – 2.8	4.1 – 10.3
Cl[e]	3.6 – 3.8	0.1 – 0.2	5.5 – 6.0
K[d]	1.4 – 3.8	0.5 – 1.3	2.9 – 7.4
Organic matter[d]	31.2 – 71.4	46.2 – 50.9	113 – 179
BOD_5[f,g]	10.3	20.3	44.7

[a]For N, P, K, and organic matter: assuming moisture content of 93-96% (Polpraset, 2007) and 1200 g urine/person/d in a rural developing country setting (Feacham et al., 1983).
[b]For N, P, K, and organic matter: assuming moisture content of 85% and 350 g wet feces/person/d in a rural developing country setting (Feacham et al., 1983).
[c]Based on 4 people per latrine.
[d]Composition data from Polpraset (2007), based on Gotaas (1956) and Feacham et al. (1983).
[e]BGS (2002).
[f]Feacham et al. (1983).
[g]BOD_5 – Biochemical Oxygen Demand-5: The amount of dissolved oxygen consumed in during wastewater decomposition in five days. This represents a measure of organic matter that can be broken down by biological processes.

Supplementary Material References

Anderson M, Bollinger D, Hagler A, Hartwell H, Rivers B, Ward K, et al. 2004. Viable but nonculturable bacteria are present in mouse and human urine specimens. Journal of Clinical Microbiology 42(2): 753-758.

BGS. 2002. Assessing Risk to Groundwater from On-site Sanitation: Scientific Review and Case Studies. (Groundwater Systems and Water Quality Programme Commissioned Report CR/02/079N). Keyworth, Nottingham:British Geological Survey.

Bhagwan JN, Still D, Buckley C, Foxon K. 2008. Challenges with up-scaling dry sanitation technologies. Water science and technology 58(1): 21-27.

Blaut M. 2002. Relationship of prebiotics and food to intestinal microflora. European journal of nutrition 41 Suppl 1: I11-16.

Buckley C, Foxon K, Brouckaert C, Rodda N, Nwaneri C, Balboni E, et al. 2008. Scientific Support for the Design and Operation of Ventilated Improved Pit Latrines (Vips) and the Efficacy of Pit Latrine Additives. KwaZulu-Nata:Water Research Commission.

Casson LW, Ritter MOD, Cossentino LM, Gupta P. 1997. Survival and recovery of seeded HIV in water and wastewater. Water Environment Research 69(2): 174-179

Chaggu E. 2003. Sustainable Environmental Protection Using Modified Pit-Latrines The Netherland: Wageningen University.

Drangert JO. 1998. Fighting the urine blindness to provide more sanitation option. Water Sa 24(2): 157-164.

Feachem RG, Bradley DJ, Garelick H, Mara DD. 1983. Sanitation and Disease: Health Aspects of Excreta and Wastewater Management. New York: John Wiley & Sons Ltd.

Fourie AB, Vanryneveld MB. 1995. The Fate in the Subsurface of Contaminants Associated with on-Site Sanitation - a Review. Water Sa 21(2): 101-111.

Gotaas HB. 1956. Composting: Sanitary Disposal and Reclamation of Organic Wastes. World Health Organization Monograph Series No. 31, Geneva. 205 p.

Jacks G, Sefe F, Carling M, Hammar M, Letsamao P. 1999. Tentative nitrogen budget for pit latrines – eastern Botswana. Environmental Geology 38(3): 199-203.

Johnson RW, Blatchley ER, Mason DR. 1994. HIV and the bloodborne pathogen regulation: Implications for the wastewater industry. Water Environment Research 66(5): 684-691

Kim MS, Park EJ, Roh SW, Bae JW. 2011. Diversity and Abundance of Single-Stranded DNA Viruses in Human Feces. Applied and Environmental Microbiology 77(22): 8062-8070.

Kramer A, Schwebke I, Kampf G. 2006. How long do nosocomial pathogens persist on inanimate surfaces? A systematic review. BMC Infectious Diseases 6(130): 1-8.

Ley RE, Peterson DA, Gordon JI. 2006. Ecological and evolutionary forces shaping microbial diversity in the human intestine. Cell 124(4): 837-848.

Mansour ME, Kotagal U, Rose B, Ho M, Brewer D, Roy-Chaudhury A, et al. 2003. Health-related quality of life in urban elementary schoolchildren. Pediatrics 111(6): 1372-1381.

Moore WEC, Moore LH. 1995. Intestinal Floras of Populations That Have a High-Risk of Colon-Cancer. Applied and Environmental Microbiology 61(9): 3202-3207.

Ozawa K, Oka T, Takeda N, Hansman GS. 2007. Norovirus infections in symptomatic and asymptomatic food handlers in Japan. Journal of Clinical Microbiology 45(12): 3996-4005.

Pedley S, Yates M, Schijven JF, West J, Howard G, Barrett M. 2006. Pathogens: Health relevance, transport and attenuation. In: Protecting Groundwater for Health: Managing the Quality of Drinking-Water Sources, (Organization WH, ed). London:WHO and IWA.

Pescod MB. 1971. Sludge Handling and Disposal in Tropical Developing Countries. J Water Pollut Con F 43(4): 555-&.

Polpraset C. 2007. Organic Waste Recycling: Technology and Management. IWA Publishing, London. 516 p.

Ramakrishna BS. 2007. The normal bacterial flora of the human intestine and its regulation. J Clin Gastroenterol 41(5): S2-S6.

Rodrigues C, Pinto D, Medeiros R. 2007. Molecular epidemiology characterization of the urinary excretion of polyomavirus in healthy individuals from Portugal - A southern European population. Journal of medical virology 79(8): 1194-1198.

Schouw NL, Danteravanich S, Mosbaek H, Tjell JC. 2002. Composition of human excreta - a case study from Southern Thailand. Science of the Total Environment 286: 155-166.

Still DA. 2002. After the Pit Latrine is Full....What Then? Effective Options for Pit Latrine Management. In: Biennial Conference of the Water Institute of Southern Africa. Durban, South Africa.

UNICEF. 2012. Multiple Indicator Cluster Surveys. Available: http://www.childinfo.org/mics.html [accessed August 24, 2012].

United Nations, Department of Economic and Social Affairs, Population Division. 2011. World Population Prospects: The 2010 Revision, CD-ROM Edition

USAID. 2012. Demographic Health Surveys. Available: http://www.measuredhs.com [accessed August 24, 2012 2012].

Wandell BA, Wade AR. 2003. Functional imaging of the visual pathways. Neurologic clinics 21(2): 417-+.

WHO. 2011. Nitrate and nitrite in drinking-water. (Background document for development of WHO Guidelines for Drinking-water Quality). Geneva, Switzerland:World Health Organization.

WHO. 2006. Protecting Groundwater for Health: Managing the Quality of Drinking-water Sources. London: World Health Organization.

WHO/UNICEF. 2012a. Economic, Population, Nutrition, and Health Survey. [Online August 24, 2012].

Zhang T, Breitbart M, Lee WH, Run JQ, Wei CL, Soh SWL, et al. 2006. RNA viral community in human feces: Prevalence of plant pathogenic viruses. PLoS biology 4(1): 108-118.
